# A Thi2p Regulatory Network Controls the Post-glucose Effect of Xylose Utilization in *Saccharomyces cerevisiae*

**DOI:** 10.3389/fmicb.2019.01649

**Published:** 2019-07-17

**Authors:** Shan Wei, Penggang Bai, Yanan Liu, Mengdan Yang, Juanzhen Ma, Jin Hou, Weifeng Liu, Xiaoming Bao, Yu Shen

**Affiliations:** ^1^State Key Laboratory of Microbial Technology, Microbiology and Biotechnology Institute, Shandong University, Qingdao, China; ^2^Shandong Provincial Key Laboratory of Microbial Engineering, Qi Lu University of Technology, Jinan, China

**Keywords:** *Saccharomyces cerevisiae*, xylose metabolism, regulation of carbon metabolism, Thi2p, anaerobic fermentation, the post-glucose effect

## Abstract

The complete and efficient utilization of both glucose and xylose is necessary for the economically viable production of biofuels and chemicals using lignocellulosic feedstocks. Although recently obtained recombinant *Saccharomyces cerevisiae* strains metabolize xylose well when xylose is the sole carbon source in the medium (henceforth referred to as “X stage”), their xylose consumption rate is significantly reduced during the xylose-only consumption phase of glucose-xylose co-fermentation (“GX stage”). This post-glucose effect seriously decreases overall fermentation efficiency. We showed in previous work that *THI2* deletion can alleviate this post-glucose effect, but the underlying mechanisms were ill-defined. In the present study, we profiled the transcriptome of a *thi2*Δ strain growing at the GX stage. Thi2p in GX stage cells regulates genes involved in the cell cycle, stress tolerance, and cell viability. Importantly, the regulation of Thi2p differs from a previous regulatory network that functions when glucose is the sole carbon source, which suggests that the function of Thi2p depends on the carbon source. Modeling research seeking to optimize metabolic engineering via TFs should account for this important carbon source difference. Building on our initial study, we confirmed that several identified factors did indeed increase fermentation efficiency. Specifically, overexpressing *STT4, RGI2*, and *TFC3* increases specific xylose utilization rate of the strain by 36.9, 29.7, 42.8%, respectively, in the GX stage of anaerobic fermentation. Our study thus illustrates a promising strategy for the rational engineering of yeast for lignocellulosic ethanol production.

## Introduction

The economic feasibility of producing biofuels and biochemicals via the industrial fermentation of lignocellulosic hydrolysates requires the full consumption of glucose and xylose, which are the most abundant sugars in this kind of material (Hou et al., 2017; Kwak et al., 2019; Li et al., 2019). *Saccharomyces cerevisiae* is a well-studied and robust cellular factory, but it cannot natively metabolize xylose. Engineering strategies have introduced the initial xylose metabolizing enzymes, the xylose isomerase (XI), or xylose reductase and xylitol dehydrogenase. Strategies have also focused on altered transporters and modified expression of genes encoding xylulokinase and non-oxidative pentose phosphate pathway (PPP) in *S. cerevisiae*. However, such recombinant strains based on the strategies mentioned above only show limited xylose utilization capacity. Additionally, directed evolution with xylose as the sole carbon source in the growth medium has led to some substantial improvements (Hou et al., 2017; Kwak et al., 2019; Li et al., 2019). However, in many cases, the mechanistic details remain unclear, and this lack of understanding has hindered progress for realizing advanced strategies to rationally engineer further improvements (Myers et al., 2019).

Factors, including metabolic genes and transcription factors (TFs), that control xylose utilization in *S. cerevisiae* have been the focus of specific research in recent decades. For example, it has been confirmed that XI has significant effects on the capacity of *S. cerevisiae* to metabolize xylose. Efficient xylose utilization by an evolved strain has been partially attributed to elevated expression levels of XI, which was accomplished via multiple-copy chromosomal integration of the heterogenous *xylA* gene (Zhou et al., 2012). The enhancement of another evolved strain was attributed to the improvement of XI activity in *S. cerevisiae* by upregulating the expression molecular chaperones (Hou et al., 2016a). Additionally, improved xylose utilization capacity in some other evolved strains was attributed to the reprogramming of their carbon metabolism regulatory networks, such as the MAP Kinase (MAPK) signaling pathway and Protein Kinase A (PKA) signaling pathways, and several TFs in these signaling pathways, such as Hog1p and Ira2p, have showed their effects on xylose metabolism (Sato et al., 2016; Osiro et al., 2018; Myers et al., 2019). These findings confirmed that globally modifying the gene expression state by regulate the key transcription factors could be a way to optimize the xylose metabolism in yeast.

The carbon source conditions at the start of fermentation also significantly affect xylose metabolism. The evolved strain metabolize xylose well when xylose is the sole carbon source (referred to as the X stage). However, their specific xylose consumption rate is generally lower in the xylose consumption phase after glucose depletion in glucose-xylose co-fermentation (referred to as the GX stage), although there still remains more than half of the xylose when cells enter the GX stage (Michael et al., 2016; Wei et al., 2018). That is the yeast cells do not recognize xylose in the GX stage as they do in the X stage. It is industrially attractive to alleviate this post-glucose effect because it significantly decreases xylose utilization and prolongs fermentation times. To date, there are insufficient data regarding the mechanisms of control of the post-glucose effect and limited strategies to overcome it.

In our previous work, we revealed that deletion of the TF gene *THI2* improved the xylose consumption in the GX stage (Wei et al., 2018). Thi2p is a transcriptional activator of thiamine biosynthetic genes (Nosaka et al., 2005), but little information exists on how Thi2p affects carbon metabolism. Here, we demonstrated that deletion of *THI2* does not affect the activity of xylose isomerase, which catalyzes the first step of xylose metabolism and significantly affects the metabolic efficiency. We then compared the specific transcriptome differences during the GX stage between the *thi2Δ* strain and parental strain and examined the effects of Thi2p target genes on xylose metabolism. We thusly discovered a Thi2p regulatory network that improved xylose utilization in the GX stage. In addition, we revealed that deleting *THI2* or overexpressing its target genes *MID2, STT4*, and *CDC42* decreased the proportion of dead cells present in cultures. Finally, we showed that overexpressing Thi2p target genes *STT4* (Phosphatidylinositol-4-kinase), *RGI2* (respiratory growth induced, function unknown), and *TFC3* (subunit of RNA polymerase III transcription initiation factor complex) significantly enhanced xylose utilization in the GX stage of anaerobic fermentation, thereby illustrating a promising strategy for the rational engineering of yeast for lignocellulosic ethanol production. Moreover, our work illustrates the important point that yeast metabolic modeling, both in basic systems studies and in more applied efforts directed towards optimization and engineering, needs to account for the carbon-source-dependent regulatory functions of TFs like Thi2p.

## Methods

### Construction of Plasmids and Strains

All plasmids and strains used in this study are listed in Table 1. The ORFs of all genes were amplified from the genomic DNA of the *S. cerevisiae* strain CEN.PK 113-5D (Entian and Kotter, 2007) using the primers listed in Additional file 1: Supplementary Table S1. The fragments of ORFs were digested by restriction enzymes and ligated into plasmid pUC20. The genes in the resultant recombinant plasmids were under the control of the *TEF1* promoter. The genes were overexpressed by transferring these recombinant plasmids into BSGX001.

**Table 1:** *S. cerevisiae* strains and plasmids used in this study.

***S. cerevisiae* strains and plasmids**	**Description**	**Sources**
**PLASMIDS**		
pUG6	The plasmid with *loxP-KanMX4-loxP* cassette	Guldener et al., 1996
YEp-CH	Shuttle plasmid for *E. coli* and *S. cerevisiae, GAL2p-cre-CYC1t, HygR*	Li et al., 2016
pUC20	Yeast 2μ plasmid, *KanMX4*	Wei et al., 2018
pUC20-*BDH2^a^*	pUC20, *TEF1p-BDH2-ADHt*	This study
***S. cerevisiae*** **STRAINS**		
CEN.PK 113-5D	*MATa; ura3-53*	Entian and Kotter, 2007
BSGX001	CEN.PK 113-5D derivative; Ru-XI, XK, *gre3*::PPP, *cox4Δ*, AE^b^	Hou et al., 2016b
BSGX001(*ixr1Δ*)^c^	BSGX001 derivative, *ixr1::KanMX4*	This study
BSGX001(*BDH2*)^d^	BSGX001 derivative, δ1-*loxp-TEF1p-BDH2-ADHt*-δ2	This study

a *Other plasmids derived from pUC20 were named in the same way, and due to space limitations, they were not listed here*.

b *AE, adaptive evolution in medium using xylose as the sole carbon source*.

c *Other strains derived from BSGX001 with deleted genes were named in the same way, and due to space limitations, they were not listed here*.

d *Other strains derived from BSGX001 with overexpressed genes were named in the same way, and due to space limitations, they were not listed here*.

Gene knockout was performed by homologous recombination using a *KanMX4* expression cassette, which was cloned from pUG6 (Guldener et al., 1996), to replace the target gene. The *KanMX4* marker was then discarded by transferring plasmid YEp-CH into the strains and inducing the expression of Cre recombinase (Li et al., 2016).

### Cultivation Conditions and Batch Fermentation

*E. coli* recombinant cells were cultured at 37°C in Luria–Bertani (LB) medium (5 g L^−1^ yeast extract, 10 g L^−1^ tryptone, 10 g L^−1^ NaCl, pH 7.0), and 100 mg L^−1^ ampicillin was added for the selection of transformants. Yeast cells were cultivated at 30 °C in SC-Ura medium containing 1.7 g L^−1^ yeast nitrogen base, 5 g L^−1^ (NH_4_)_2_SO_4_, 0.77 g L^−1^ CSM-Ura (Sunrise Science Products, USA) and 20 g L^−1^ glucose as the carbon source.

Fermentation was performed in shake flasks or 1 L bioreactors according to the experimental requirements. The fermentation medium was comprised of 1.7 g L^−1^ yeast nitrogen base, 5 g L^−1^ (NH_4_)_2_SO_4_, 20 g L^−1^ glucose, and 20 g L^−1^ xylose. Overnight cultures of a single colony were transferred into a 250 mL shake flask containing 50–60 mL fresh SC-Ura medium supplied with 20 g L^−1^ glucose and an initial biomass of 0.23 g L^−1^ dry cell weight (DCW) (OD_600_ of 1) and cultured at 30 °C and 200 rpm for another 12–16 h. The cells were then collected and washed three times with sterile water and inoculated into the fermentation medium. Fermentation in shake flasks was performed at 30°C and 200 rpm. The initial biomass was 0.575 g L^−1^ DCW. The anaerobic fermentation in bioreactors was performed with an initial biomass of 0.23 g L^−1^ DCW at 30°C and pH 5.5, with 0.1 vvm nitrogen and a stirring speed of 200 rpm for ~ 30 h. The pH was maintained by automatically pumping 5 mol L^−1^ NaOH and 5 mol L^−1^ H_3_PO_4_. All fermentations were carried out in triplicate.

### Quantitative PCR (qPCR) Analysis

qPCR data were analyzed according to the 2^−ΔΔ*CT*^ method (Livak and Schmittgen, 2001). RNA was extracted from the cells collected from the 20 h glucose-xylose co-fermentation flasks using a UNlQ-10 Column Trizol Total RNA Isolation Kit (Sangon Biotech Co., Ltd., Shanghai, China). The cDNA was obtained using a PrimeScript^TM^ RT reagent Kit (TaKaRa, Japan). The gene transcription levels were determined using the equation *N* = 2^Ct(reference gene)^/2^Ct(target gene)^. *ACT1* was used as the reference gene, and the *t*-test was applied to evaluate the differences between means.

### Xylose Isomerase Activity Assay

The crude enzyme samples were prepared as previously described (Hou et al., 2016a). Yeast cells were collected at 20 h in glucose-xylose co-fermentation, then were broken by glass beads (Φ = 0.5 mm) using a FastPrep cell homogenizer (Thermo Savant, USA). The total cellular protein concentration was measured using a BCA protein assay reagent kit (Sangon Biotech Co., Ltd., Shanghai, China).

The XI activities were determined at 30°C by measuring the decrease in NADH concentration using a previously reported method (Hou et al., 2016a). Briefly, assays were performed in reaction mixtures containing 0.15 mmol L^−1^ NADH, 10 mmol L^−1^ MgCl_2_, and 1 U of sorbitol dehydrogenase (Sigma-Aldrich, USA) in 100 mmol L^−1^ Tris-HCl (pH 7.5) with appropriately diluted crude cell extracts. The reaction was initiated by adding 500 mmol L^−1^ xylose. One unit of XI activity was defined as the amount of crude enzyme required to produce 1 mmol xylulose per min under the assay conditions.

### Transcriptome Analysis

Samples from the batch fermentation shake flasks were taken at 20 h and subjected to transcriptome analysis. The cells in each sample were collected by centrifugation at 5,000 rpm and 4°C for 5 min and then frozen in liquid nitrogen. Total RNA was extracted using a UNIQ-10 Trizol RNA Purification Kit (Sangon Biotech, China) and then fragmented. DNA was digested with DNase I, and cDNA was synthesized by using short mRNA fragments as templates. Three independent RNA extractions were assayed for each strain. The resulting sample library was sequenced using an Illumina HiSeqTM 2000 (BGI Shenzhen, China).

Raw data from transcriptional analysis and processed data for genes exhibiting significant differences between BSGX001 (*thi2*Δ) and BSGX001 are available in the NCBI Gene Expression Omnibus database (GEO Accession Number: GSE119333). Significant differences were indicated by *p*-values of 0.001 or less, and an absolute fold-change threshold of 2.0 or greater. All annotations were derived from the Saccharomyces Genome Database (SGD) (http://www.yeastgenome.org/). Cluster analysis was performed using the Gene Ontology Slim Mapper tool supplied by the SGD (http://www.yeastgenome.org).

### Analysis of Metabolites and Calculation

The concentrations of glucose, xylose, glycerol, acetate, and ethanol were measured using HPLC (Shimadzu, Japan) with an Aminex HPX-87H ion exchange column (300 ×7.8 mm, Bio-Rad, Hercules, USA). The mobile phase was 5 mmol L^−1^ H_2_SO_4_ with a flow rate of 0.6 ml/min, and the temperature of the column oven was 45°C. The specific xylose utilization rate (*r*_xylose_) was calculated using the following equation, as previously described (Wei et al., 2018):

(1) $$\begin{array}{l} r=\frac{A_{n}-A_{m}}{\frac{1}{2}\sum_{i=m+1}^{n}\left(B_{i}+B_{i-1}\right)\times \left(t_{i}-t_{i-1}\right)} \end{array}$$

where *r* is the specific utilization during the phase from sampling point *m* to sampling point *n*; and *A, B*, and *t* are the metabolite concentration, biomass concentration, and time, respectively, at sampling points *n, i*, and *m*.

### Measurement of the Proportion of Dead Cells in Culture

Yeast cells were harvested at 20, 36, and 48 h, and diluted to a suitable multiple (~ 6 ×10^7^ cells mL^−1^). The cells were then incubated with 0.04% trypan blue for 3–10 min; dead cells were stained by trypan, while live cells were not (Bowey-Dellinger et al., 2017). The stained cells were counted manually using a hemocytometer. Samples were subjected to independent triplicate tests, each with more than 500 cells counted. For statistical analysis, the unpaired, two-tailed *t*-test was performed. Data with p ≤ 0.05 were considered significantly different.

## Results

### Increased Xylose Utilization of the *THI2* Deletion Strain Is Not Related to Xylose Isomerase Activity

**Figure 1:**
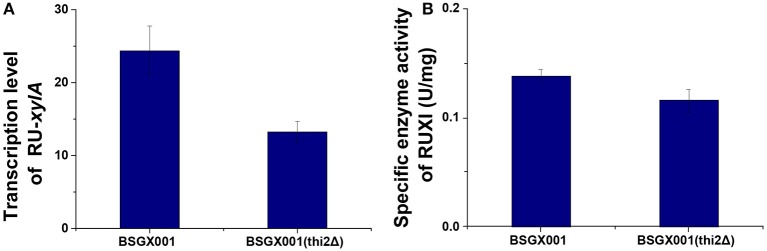
The transcription level of the Ru-*xylA* gene relative to reference gene *ACT1* **(A)**, and the specific activity of XI **(B)** of the ***THI2*** deletion strain. The error bars indicate the standard deviation of independent experiments performed in triplicate.

Xylose isomerase activity in recombinant yeast seriously affects the capacity of *S. cerevisiae* to utilize xylose (van Maris et al., 2007; Zhou et al., 2012; Hou et al., 2016a). The transcription level of *xylA* and XI activity of the *thi2*Δ strain and its control BSGX001 (Hou et al., 2016b) were detected to determine whether deletion of *THI2* enhanced xylose utilization through increasing the *xylA* gene expression or directly improving the XI activity. The results showed that the transcriptional level of *xylA* in *THI2* deletion strain is 54.2% of that in parent strain and the p-value is 0.147, which change is not significant. The xylose isomerase in *THI2* deletion strain is 83.6% of parent strain (*p*-value is 0.048). These results suggested that neither the transcription level of *xylA* nor XI activity increased in the *thi2*Δ strain compared to BSGX001 in the GX stage (Figure 1), that is deletion of *THI2* did not increase XI activity of strain.

### The Transcriptional Profile of the *THI2* Deletion Strain Suggested Engineering Strategies for Enhanced Xylose Utilization in *S. cerevisiae*

#### Transcriptional Profile of the *THI2* Deletion Strain

To investigate how *THI2* deletion improved the xylose utilization in the GX stage, we compared the transcriptome of *THI2* deletion strain BSGX001(*thi2*Δ) and its parent strain BSGX001 in the GX stage. The samples of both strains were taken at 2 h after glucose depletion (20 h) in glucose-xylose co-fermentation. At this time point, ~17–18 g L^−1^ xylose remained in the medium. The transcriptome analysis results (Table 2) revealed that 93 and 16 genes were significantly up- and downregulated, respectively, in the *THI2* deletion strain during the GX stage. The Gene Ontology (GO) cluster result showed that within the Molecular Functions category, the upregulated genes were primarily clustered (cluster frequency ≥ 10%) under the GO terms hydrolase activity and DNA binding; the downregulated genes were primarily clustered under the GO terms DNA binding, transferase activity and ligase activity; within the Biological Processes category, upregulated genes did not cluster to specific GO term, while the downregulated genes clustered to GO terms RNA polymerase II promoter, cellular response to DNA damage stimulus, vitamin metabolic process, and DNA repair.

**Table 2:** Gene cluster analysis of transcriptome difference of BSGX001 (thi2Δ) vs. BSGX001 at the GX stage in the aspect of Molecular Function and Biological Processes.

**GOID**	**GO terms (Molecular Function)**	**Frequency**	**Gene(s)**
**UP-REGULATED**			
3674	Molecular function unknown	34 out of 93 genes, 36.6%	*ATS1, MAK16, BOL1, BOL3, AIM2, ECM1, ERP1, PAU7, YAR023C, UIP3, MST28, YAR064W, YAR066W, YAR068W, RRT6, SPG1, RGI2, FAR10, ARV1, SYM1, YLR255C, TMA7, YLR264C-A, BOP2, CMG1, SMD2, YLR281C, YLR283W, YLR287C, COQ11, SPH1, NKP2, PEX30, YLR326W*
16787	Hydrolase activity	14 out of 93 genes, 15.1%	*CCR4, POP5, PHO11, YIL082W-A, CDC42, GPN3, CDD1, IRC20, MCM5, DBP9, CTS1, TAD3, SFH1, YRF1-6*
3677	DNA binding	11 out of 93 genes, 11.8%	*TFC3, SAW1, ECM22, EST1, RED1, NEJ1, PDR8, MCM5, YLR278C, MEC3, EST2*
16740	Transferase activity	9 out of 93 genes, 9.7%	*SWD1, YAT1, YIL082W-A, ERF2, IRC20, STT4, UBC12, EST2, GAS2*
30234	Enzyme regulator activity	8 out of 93 genes, 8.6%	*GIP4, CLN3, PEX22, GPB2, BUD14, RFU1, PIG1, GCD7*
5198	Structural molecule activity	7 out of 93 genes, 7.5%	*NUP60, RED1, RPS28B, MRPL15, RPL38, RPS25B, RPP0*
3723	RNA binding	7 out of 93 genes, 7.5%	*POP5, YIL082W-A, EST1, DBP9, GCD7, YHC1, RPP0*
3735	Structural constituent of ribosome	5 out of 93 genes, 5.4%	*RPS28B, MRPL15, RPL38, RPS25B, RPP0*
4386	Helicase activity	4 out of 93 genes, 4.3%	*IRC20, MCM5, DBP9, YRF1-6*
16887	ATPase activity	3 out of 93 genes, 3.2%	*MCM5, DBP9, SFH1*
4518	Nuclease activity	3 out of 93 genes, 3.2%	*CCR4, POP5, YIL082W-A*
1071	Nucleic acid binding transcription factor activity	3 out of 93 genes, 3.2%	*TFC3, ECM22, PDR8*
3682	Chromatin binding	3 out of 93 genes, 3.2%	*RED1, YCS4, MCM5*
5085	Guanyl-nucleotide exchange factor activity	3 out of 93 genes, 3.2%	*EFB1, LTE1, GCD7*
19899	Enzyme binding	2 out of 93 genes, 2.2%	*VPS8, GIP4*
3729	mRNA binding	2 out of 93 genes, 2.2%	*DBP9, YHC1*
16779	nucleotidyltransferase activity	2 out of 93 genes, 2.2%	*YIL082W-A, EST2*
4871	Signal transducer activity	2 out of 93 genes, 2.2%	*GPB2, MID2*
43167	Ion binding	2 out of 93 genes, 2.2%	*RBG1, NUP60*
3924	GTPase activity	2 out of 93 genes, 2.2%	*CDC42, GPN3*
22857	Transmembrane transporter activity	2 out of 93 genes, 2.2%	*THI7, CSC1*
8135	Translation factor activity, RNA binding	1 out of 93 genes, 1.1%	*GCD7*
16491	Oxidoreductase activity	1 out of 93 genes, 1.1%	*BDH2*
16798	Hydrolase activity, acting on glycosyl bonds	1 out of 93 genes, 1.1%	*CTS1*
8233	Peptidase activity	1 out of 93 genes, 1.1%	*YIL082W-A*
16853	Isomerase activity	1 out of 93 genes, 1.1%	*ECI1*
16301	Kinase activity	1 out of 93 genes, 1.1%	*STT4*
988	Transcription factor activity, protein binding	1 out of 93 genes, 1.1%	*TFC3*
30674	Protein binding, bridging	1 out of 93 genes, 1.1%	*ATG39*
19843	rRNA binding	1 out of 93 genes, 1.1%	*RPP0*
8168	Methyltransferase activity	1 out of 93 genes, 1.1%	*SWD1*
8289	Lipid binding	1 out of 93 genes, 1.1%	*NUP60*
16829	Lyase activity	1 out of 93 genes, 1.1%	*CYC3*
16874	Ligase activity	1 out of 93 genes, 1.1%	*LIP2*
51082	Unfolded protein binding	1 out of 93 genes, 1.1%	*CNE1*
16791	Phosphatase activity	1 out of 93 genes, 1.1%	*PHO11*
**DOWN-REGULATED**			
3674	Molecular function unknown	6 out of 16 genes, 37.5%	*BSC1, PRM7, YDR246W-A, RRT5, YGL015C, YOR338W*
3677	DNA binding	3 out of 16 genes, 18.8%	*THI2, MGA1, IXR1*
16740	Transferase activity	3 out of 16 genes, 18.8%	*HOM3, TRA1, URA2*
16874	Ligase activity	2 out of 16 genes, 12.5%	*SNZ3, URA2*
30234	Enzyme regulator activity	1 out of 16 genes, 6.3%	*CIP1*
1071	Nucleic acid binding transcription factor activity	1 out of 16 genes, 6.3%	*MGA1*
16829	Lyase activity	1 out of 16 genes, 6.3%	*DAL3*
16301	Kinase activity	1 out of 16 genes, 6.3%	*HOM3*
other	Other	1 out of 16 genes, 6.3%	*BTN2*
**GOID**	**GO terms (Biological Processes)**	**Frequency**	**Gene(s)**
**UP-REGULATED**			
8150	Biological process unknown	19 out of 93 genes, 20.4%	*AIM2, BDH2, PAU7, YAR023C, UIP3, YAR064W, YAR066W, YAR068W, RRT6, SPG1, YLR255C, YLR264C-A, BOP2, CMG1, YLR278C, YLR281C, YLR283W, YLR287C, YLR326W*
51726	Regulation of cell cycle	9 out of 93 genes, 9.7%	*CCR4, LTE1, CLN3, BUD14, CDC42, RED1, YCS4, MEC3, SFH1*
33043	Regulation of organelle organization	8 out of 93 genes, 8.6%	*EFB1, LTE1, BUD14, CDC42, EST1, RED1, YCS4, MCM5*
6974	Cellular response to DNA damage stimulus	7 out of 93 genes, 7.5%	*SAW1, NUP60, IRC20, NEJ1, MCM5, MEC3, SFH1*
7059	Chromosome segregation	7 out of 93 genes, 7.5%	*LTE1, GIP4, GPN3, RED1, YCS4, NKP2, SFH1*
278	Mitotic cell cycle	7 out of 93 genes, 7.5%	*CCR4, LTE1, CLN3, CDC42, GPN3, YCS4, SPH1*
42221	Response to chemical	6 out of 93 genes, 6.5%	*GPB2, CNE1, CDC42, FAR10, SPH1, MID2*
6281	DNA repair	6 out of 93 genes, 6.5%	*SAW1, NUP60, IRC20, NEJ1, MCM5, SFH1*
6325	Chromatin organization	6 out of 93 genes, 6.5%	*NUP60, SWD1, YCS4, MCM5, MEC3, SFH1*
2181	Cytoplasmic translation	6 out of 93 genes, 6.5%	*RBG1, TMA7, RPS28B, RPL38, RPS25B, RPP0*
6605	Protein targeting	5 out of 93 genes, 5.4%	*VPS8, PEX22, NUP60, GPN3, ERF2*
51052	Regulation of DNA metabolic process	5 out of 93 genes, 5.4%	*CCR4, SAW1, EST1, MCM5, SFH1*
6366	Transcription from RNA polymerase II promoter	5 out of 93 genes, 5.4%	*CCR4, GPB2, ECM22, PDR8, SFH1*
48285	Organelle fission	5 out of 93 genes, 5.4%	*LTE1, CDC42, GPN3, RED1, YCS4*
23052	Signaling	5 out of 93 genes, 5.4%	*PEX22, GPB2, CDC42, FAR10, MID2*
32200	Telomere organization	5 out of 93 genes, 5.4%	*SWD1, EST1, MEC3, EST2, YRF1-6*
7010	Cytoskeleton organization	4 out of 93 genes, 4.3%	*EFB1, ATS1, BUD14, CDC42*
746	Conjugation	4 out of 93 genes, 4.3%	*CDC42, FAR10, SPH1, MID2*
31399	Regulation of protein modification process	4 out of 93 genes, 4.3%	*GIP4, CLN3, PEX22, NUP60*
6629	Lipid metabolic process	4 out of 93 genes, 4.3%	*ECM22, ARV1, ECI1, STT4*
902	Cell morphogenesis	4 out of 93 genes, 4.3%	*BUD14, CDC42, SPH1, MID2*
6310	DNA recombination	4 out of 93 genes, 4.3%	*IRC20, MCM5, MEC3, YRF1-6*
51169	Nuclear transport	4 out of 93 genes, 4.3%	*ECM1, NUP60, GPN3, RPS28B*
51321	Meiotic cell cycle	4 out of 93 genes, 4.3%	*GPB2, RED1, YCS4, GAS2*
6397	mRNA processing	3 out of 93 genes, 3.2%	*SMD2, YHC1, MID2*
8380	RNA splicing	3 out of 93 genes, 3.2%	*SMD2, YHC1, MID2*
6401	RNA catabolic process	3 out of 93 genes, 3.2%	*CCR4, POP5, RPS28B*
6260	DNA replication	3 out of 93 genes, 3.2%	*CCR4, MCM5, SFH1*
7114	Cell budding	3 out of 93 genes, 3.2%	*ATS1, CDC42, SPH1*
8033	tRNA processing	3 out of 93 genes, 3.2%	*ATS1, POP5, TAD3*
7124	Pseudohyphal growth	3 out of 93 genes, 3.2%	*GPB2, CDC42, SPH1*
51049	Regulation of transport	3 out of 93 genes, 3.2%	*BUD14, ECM22, CDC42*
6364	rRNA processing	3 out of 93 genes, 3.2%	*MAK16, POP5, DBP9*
70647	Protein modification by small protein conjugation or removal	3 out of 93 genes, 3.2%	*PEX22, NUP60, UBC12*
42273	Ribosomal large subunit biogenesis	3 out of 93 genes, 3.2%	*MAK16, DBP9, RPP0*
9451	RNA modification	2 out of 93 genes, 2.2%	*ATS1, TAD3*
6497	Protein lipidation	2 out of 93 genes, 2.2%	*ARV1, ERF2*
6869	Lipid transport	2 out of 93 genes, 2.2%	*ECM22, ARV1*
55085	Transmembrane transport	2 out of 93 genes, 2.2%	*PEX22, THI7*
70271	Protein complex biogenesis	2 out of 93 genes, 2.2%	*CYC3, BUD14*
18193	Peptidyl-amino acid modification	2 out of 93 genes, 2.2%	*NUP60, SWD1*
42594	Response to starvation	2 out of 93 genes, 2.2%	*RBG1, ECM22*
71554	Cell wall organization or biogenesis	2 out of 93 genes, 2.2%	*MID2, GAS2*
910	Cytokinesis	2 out of 93 genes, 2.2%	*BUD14, SPH1*
7033	Vacuole organization	2 out of 93 genes, 2.2%	*CLN3, CDC42*
6091	Generation of precursor metabolites and energy	2 out of 93 genes, 2.2%	*RGI2, PIG1*
43934	Sporulation	2 out of 93 genes, 2.2%	*GPB2, GAS2*
15931	Nucleobase-containing compound transport	2 out of 93 genes, 2.2%	*NUP60, RPS28B*
51186	Cofactor metabolic process	2 out of 93 genes, 2.2%	*BOL1, COQ11*
6354	DNA-templated transcription, elongation	2 out of 93 genes, 2.2%	*CCR4, SFH1*
51604	Protein maturation	2 out of 93 genes, 2.2%	*BOL1, BOL3*
1403	Invasive growth in response to glucose limitation	2 out of 93 genes, 2.2%	*GPB2, CDC42*
7031	Peroxisome organization	2 out of 93 genes, 2.2%	*PEX22, PEX30*
6417	Regulation of translation	2 out of 93 genes, 2.2%	*EFB1, GCD7*
48284	Organelle fusion	2 out of 93 genes, 2.2%	*CLN3, CDC42*
61025	Membrane fusion	2 out of 93 genes, 2.2%	*CLN3, CDC42*
5975	Carbohydrate metabolic process	1 out of 93 genes, 1.1%	*PIG1*
6413	Translational initiation	1 out of 93 genes, 1.1%	*GCD7*
16570	Histone modification	1 out of 93 genes, 1.1%	*SWD1*
6468	Protein phosphorylation	1 out of 93 genes, 1.1%	*CLN3*
16197	Endosomal transport	1 out of 93 genes, 1.1%	*VPS8*
70925	Organelle assembly	1 out of 93 genes, 1.1%	*RPP0*
32196	Transposition	1 out of 93 genes, 1.1%	*YIL082W-A*
6887	Exocytosis	1 out of 93 genes, 1.1%	*CDC42*
6970	Response to osmotic stress	1 out of 93 genes, 1.1%	*MID2*
43144	snoRNA processing	1 out of 93 genes, 1.1%	*POP5*
54	Ribosomal subunit export from nucleus	1 out of 93 genes, 1.1%	*ECM1*
8213	Protein alkylation	1 out of 93 genes, 1.1%	*SWD1*
6457	Protein folding	1 out of 93 genes, 1.1%	*CNE1*
6383	Transcription from RNA polymerase III promoter	1 out of 93 genes, 1.1%	*TFC3*
42255	Ribosome assembly	1 out of 93 genes, 1.1%	*RPP0*
6414	Translational elongation	1 out of 93 genes, 1.1%	*EFB1*
7005	Mitochondrion organization	1 out of 93 genes, 1.1%	*STT4*
32543	Mitochondrial translation	1 out of 93 genes, 1.1%	*MRPL15*
32787	Monocarboxylic acid metabolic process	1 out of 93 genes, 1.1%	*ECI1*
16050	Vesicle organization	1 out of 93 genes, 1.1%	*MST28*
51603	Proteolysis involved in cellular protein catabolic process	1 out of 93 genes, 1.1%	*CNE1*
6811	Ion transport	1 out of 93 genes, 1.1%	*CSC1*
6470	Protein dephosphorylation	1 out of 93 genes, 1.1%	*GIP4*
43543	Protein acylation	1 out of 93 genes, 1.1%	*ERF2*
55086	Nucleobase-containing small molecule metabolic process	1 out of 93 genes, 1.1%	*CDD1*
48193	Golgi vesicle transport	1 out of 93 genes, 1.1%	*ERP1*
6897	Endocytosis	1 out of 93 genes, 1.1%	*ARV1*
9408	Response to heat	1 out of 93 genes, 1.1%	*NUP60*
other	other	7 out of 93 genes, 7.5%	*YAT1, PHO11, RFU1, LIP2, SYM1, CTS1, ATG39*
**DOWN-REGULATED**			
6366	Transcription from RNA polymerase II promoter	4 out of 16 genes, 25%	*THI2, TRA1, IXR1, YOR338W*
8150	Biological process unknown	4 out of 16 genes, 25%	*BSC1, YDR246W-A, RRT5, YGL015C*
6520	Cellular amino acid metabolic process	2 out of 16 genes, 12.5%	*HOM3, URA2*
6974	Cellular response to DNA damage stimulus	2 out of 16 genes, 12.5%	*TRA1, IXR1*
6766	Vitamin metabolic process	2 out of 16 genes, 12.5%	*THI2, SNZ3*
6281	DNA repair	2 out of 16 genes, 12.5%	*TRA1,IXR1*
6468	Protein phosphorylation	1 out of 16 genes, 6.3%	*CIP1*
16197	Endosomal transport	1 out of 16 genes, 6.3%	*BTN2*
43543	Protein acylation	1 out of 16 genes, 6.3%	*TRA1*
6457	Protein folding	1 out of 16 genes, 6.3%	*BTN2*
55086	Nucleobase-containing small molecule metabolic process	1 out of 16 genes, 6.3%	*URA2*
51726	Regulation of cell cycle	1 out of 16 genes, 6.3%	*CIP1*
42221	Response to chemical	1 out of 16 genes, 6.3%	*IXR1*
43934	Sporulation	1 out of 16 genes, 6.3%	*YOR338W*
18193	Peptidyl-amino acid modification	1 out of 16 genes, 6.3%	*TRA1*
51321	Meiotic cell cycle	1 out of 16 genes, 6.3%	*YOR338W*
6865	Amino acid transport	1 out of 16 genes, 6.3%	*BTN2*
746	Conjugation	1 out of 16 genes, 6.3%	*PRM7*
31399	Regulation of protein modification process	1 out of 16 genes, 6.3%	*CIP1*
278	Mitotic cell cycle	1 out of 16 genes, 6.3%	*CIP1*
16570	Histone modification	1 out of 16 genes, 6.3%	*TRA1*
6325	Chromatin organization	1 out of 16 genes, 6.3%	*TRA1*
6811	Ion transport	1 out of 16 genes, 6.3%	*BTN2*
other	Other	2 out of 16 genes, 12.5%	*MGA1, DAL3*

#### The Effect of Differentially Expressed Genes (DEGs) on Xylose Utilization

According to the significant DEGs and previously reported factors that related to the xylose metabolism of *S. cerevisiae* (Salusjarvi et al., 2008; Cheng et al., 2018; Wei et al., 2018), 32 upregulated genes involved in ribosomal biosynthesis, signal transducer, generation of precursor metabolites and energy, starvation response, ATPase or GTPase activity, oxidoreductase activity, cofactor metabolic process, lipid metabolic process, transmembrane transporter activity, and protein modification, and 12 downregulated genes, were chosen for follow-up investigations.

**Figure 2:**
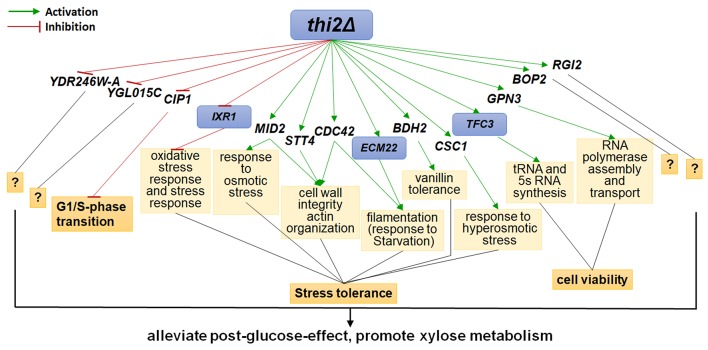
Regulatory Network of Thi2p promoting xylose utilization in the GX stage. Green and red lines represent activation function and inhibition function, respectively; black lines represent unknown function. Genes with purple background frame encode transcription factors.

The up- and down-regulated genes were overexpressed and deleted, respectively, in strain BSGX001. The effect was then evaluated by determining the xylose-specific consumption rate of recombinant strains in shake flask fermentations at the GX stage. The results showed that all mutants had no significant effect on glucose metabolism. Overexpressing the cell wall integrity (CWI)-related genes *MID2, STT4*, and *CDC42* increased the r_xylose_ of the strain by 45.9, 49.2, and 13.1%, respectively. Overexpressing stress response genes *ECM22, CSC1*, and *BDH2* increased the r_xylose_ of the strain by 11.5, 13.1, and 26.2%, respectively. Overexpressing *GPN3* (encoding a putative GTPase) and *TFC3* (encoding a subunit of the RNA polymerase III transcription initiation factor complex) increased the r_xylose_ of the strain by 13.1 and 42.6%, respectively. Furthermore, overexpressing the function unknown genes *BOP2* and *RGI2* increased the r_xylose_ of the strain by 11.5 and 41.0%, respectively. Deleting *CIP1, IXR1, YDR246W-A*, and *YGLO15C* increased the r_xylose_ of the strain by 26.2%, 36.1, 16.4, and 14.8%, respectively (Table 3). These results suggested that deleting *THI2* enhanced xylose utilization through regulating these genes (Figure 2).

**Table 3:** Genes regulated by Thi2p in GX stage and their effects on xylose utilization.

**Category**	**Strains**	**Gene annotation**	**Log_2_(fold changes)^a^**	**${\text{r}}_{\text{xylose}}^{\text{b}}$ (g g^**−1**^ DCW h^**−1**^)**
	Control (BSGX001)			0.061 ± 0.001
**OVEREXPRESSION THE UP-REGULATED GENES**				
RP-related genes	*RPS25B*	Protein component of the small (40S) ribosomal subunit	1.379	0.053 ± 0.001^*^
	*MRPL15*	Mitochondrial ribosomal protein of the large subunit	1.157	0.035 ± 0.003^*^
	*RPL38*	Ribosomal 60S subunit protein L38	1.048	0.022 ± 0.001^*^
	*MAK16*	Constituent of 66S pre-ribosomal particles	1.802	0.052 ± 0.001^*^
	*RPPO*	Conserved ribosomal protein P0 of the ribosomal stalk	1.345	0.054 ± 0.001^*^
	*RPS28B*	Protein component of the small (40S) ribosomal subunit	1.113	0.060 ± 0.000^*^
	*DBP9*	A putative ATP-dependent RNA helicase involved in 60S-ribosomal-subunit biogenesis	1.380	0.060 ± 0.000^*^
	*POP5*	Subunit of both RNase MRP and nuclear RNase P	1.616	0.050 ± 0.001^*^
	*RBG1*	Translating ribosomes	1.275	0.060 ± 0.001^*^
Signal transducer genes	*GPB2*	Multistep regulator of cAMP-PKA signaling	1.146	0.039 ± 0.003^*^
	***MID2***	Acts as a sensor for cell wall integrity signaling	**1.053**	0.089 ± 0.003^*^
	***CDC42***	Establishment and maintenance of cell polarity	**1.241**	0.069 ± 0.002^*^
	*PEX22*	Required for import of peroxisomal proteins	1.422	0.049 ± 0.001^*^
	*FAR10*	Protein involved in recovery from arrest in response to pheromone	1.051	0.060 ± 0.001^*^
Generation of precursor Metabolites and energy	*PIG1*	Glycogen synthesis	1.412	0.045 ± 0.001^*^
	***RGI2***	Involved in energy metabolism under respiratory conditions	**1.136**	0.086 ± 0.001^*^
Response to starvation	***ECM22***	Sterol regulatory element binding protein	**1.268**	0.068 ± 0.002^*^
ATPase activity	*MCM5*	An active ATP-dependent helicase	1.334	0.022 ± 0.000^*^
	*SFH1*	Component of the RSC chromatin remodeling complex	1.075	0.000 ± 0.000^*^
GTPase activity	***GPN3***	Biogenesis of RNA pol II and polIII	**1.252**	0.069 ± 0.001^*^
Oxidoreductase activity	***BDH2***	Putative medium-chain alcohol dehydrogenase	**1.241**	0.077 ± 0.001^*^
Transcription factor activity, protein binding	***TFC3***	Subunit of RNA polymerase III Transcription initiation factor complex	**1.014**	0.087 ± 0.000^*^
Cofactor metabolic process	***COQ11***	Putative oxidoreductase, subunit of Coenzyme Q biosynthetic complexes	**1.069**	**0.064 ± 0.000**
	*BOL1*	Mitochondrial matrix protein involved in Fe-S cluster biogenesis	1.014	0.060 ± 0.002
Lipid metabolic process	***STT4***	Phosphatidylinositol-4-kinase	**1.282**	0.091 ± 0.001^*^
	*ARV1*	Involved in intracellular sterol and sphingolipid transport	1.374	0.055 ± 0.003^*^
	*ECI1*	Essential for the beta-oxidation of unsaturated fatty acids	1.306	0.057 ± 0.000^*^
Transmembrane Transporter activity	***CSC1***	May be involved in detoxification	**1.071**	0.069 ± 0.002^*^
	*THI7*	Responsible for the uptake of thiamine	1.233	0.020 ± 0.001^*^
Phosphatase activity	*PHO11*	One of three repressible acid phosphatases	1.492	0.034 ± 0.002^*^
Protein modification	*UBC12*	Related to E2 ubiquitin-conjugating enzymes	1.696	0.046 ± 0.002^*^
Function unknown	***BOP2***	Protein of unknown function	**1.882**	0.068 ± 0.001^*^
**DELETION THE DOWN-REGULATED GENES**				
Cellular amino acid metabolic or transport	*hom3Δ*	Cytoplasmic enzyme that catalyzes the first step in the common pathway for methionine and threonine biosynthesis	−1.071	0.000 ± 0.000^*^
Amino acid transport	*btn2Δ*	Modulates arginine uptake	−1.038	0.030 ± 0.001^*^
Cell cycle related genes	***cip1Δ***	Cyclin-dependent kinase inhibitor	**–1.035**	0.077 ± 0.002^*^
	*yor338wΔ*	Putative protein of unknown function	−1.455	0.039 ± 0.002^*^
Response to chemical	***Ixr1Δ***	Transcriptional repressor that regulates hypoxic genes during normoxia	**–1.044**	0.083 ± 0.003^*^
DNA binding	*mga1Δ*	Protein similar to heat shock transcription factor	−1.055	0.039 ± 0.002^*^
	*dal3Δ*	Ureidoglycolate lyase	−1.108	0.037 ± 0.001^*^
Molecular function Unknown	*prm7Δ*	Pheromone-regulated protein	−1.294	0.048 ± 0.001^*^
	***ydr246w-AΔ***	Unknown function	**–1.070**	0.071 ± 0.001^*^
	*rrt5 Δ*	Unknown function	−1.004	0.051 ± 0.002^*^
	***ygl015cΔ***	Null mutants accumulate cargo in the Golgi	**–1.121**	0.070 ± 0.005^*^
	*Bsc1*	Null mutant has increased glycogen accumulation	−1.397	0.056 ± 0.004^*^

*Cells were cultured at 30°C in a shake flask and agitated at 200 rpm. All the data are the mean value ± standard deviation of independent triplicate tests. The bold gene names and values refers to the operations brought the positive effect in xylose utilization in the GX stage*.

* *p <0.05*.

a *The THI2 deletion strain compared to the parent strain BSGX001, up-regulate represents genes with higher expression in THI2 deletion strain compared to the parent strain BSGX001, down-regulate represents the reverse operation*.

*^b^The specific consumption/production rates of xylose/ethanol (r_xylose_/r_ethanol_) were calculated from the data on the xylose consumption phase in the GX stage*.

#### Overexpressing Cell Wall Integrity Related Genes *MID2, STT4*, and *CDC42* Decreased the Proportion of Dead Cells in the Culture

Among the genes that positively enhanced xylose utilization, the genes *MID2, STT4*, and *CDC42* belong to the CWI pathway, which may protect cells from environmental conditions that otherwise induced death (Mishra et al., 2017). We determined the proportion of dead cells in the culture of strains overexpressing these genes, as well as *THI2* deletion strain, and their parent strain BSGX001. Samples were taken at 20, 36, and 48 h, respectively.

**Figure 3:**
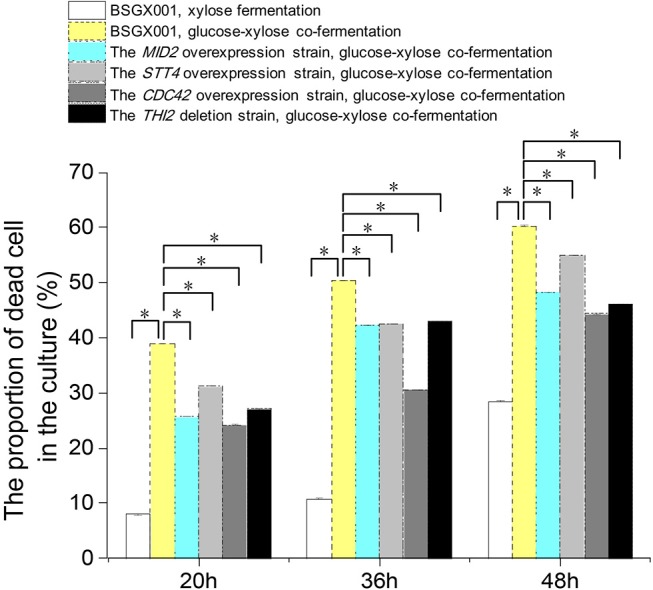
The proportion of dead cell in the culture. Cells were cultured at 30°C in a shake flask and agitated at 200 rpm. In xylose fermentation, xylose was depleted at about 30 h. In glucose-xylose co-fermentation, glucose was depleted before 20 h, while xylose was not depleted before 48 h. Samples were taken at 20, 36, and 48 h respectively. All data are the mean ± standard deviation of independent tests performed in triplicate. ^*^p < 0.05. 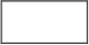, BSGX001, xylose fermentation; 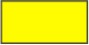, BSGX001, glucose-xylose co-fermentation; 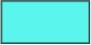, the *MID2* overexpression strain, glucose-xylose co-fermentation; 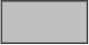, the *STT4* overexpression strain, glucose-xylose co-fermentation; 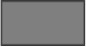, the *CDC42* overexpression strain, glucose-xylose co-fermentation; 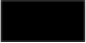, the *THI2* deletion strain, glucose-xylose co-fermentation.

The results (Figure 3) provided information regarding three aspects of xylose utilization. First, in the phase that xylose was rapidly consumed (BSGX001, xylose fermentation, 20 h), the proportion of dead cells in the culture was low (<10%). After xylose was depleted (BSGX001, xylose fermentation, 36 and 48 h), the proportion of dead cells increased with time. The changes in nutritional condition apparently induced cell death. Second, the proportion of dead cells in the GX stage (BSGX001, glucose-xylose co-fermentation, 20, 36, 48 h) was much higher than after xylose was depleted (BSGX001, xylose fermentation, 36 and 48 h), which suggested that cell death was more significantly induced by glucose depletion than by xylose depletion. Third, overexpressing *MID2, STT4*, and *CDC42* or deleting *THI2* decreased the proportion of dead cells in the cultures. The decrease at all timepoints was significant (*p*-value < 0.05). Overexpression of *MID2, STT4*, and *CDC42* or deleting *THI2* enhanced xylose metabolism in the GX stage, in part, because overexpression of these genes promoted cell survival and continued metabolism.

### Overexpressing *STT4, RGI2*, or *TFC3* Enhanced Xylose Utilization in the GX Stage Under Anaerobic Conditions

**Figure 4:**
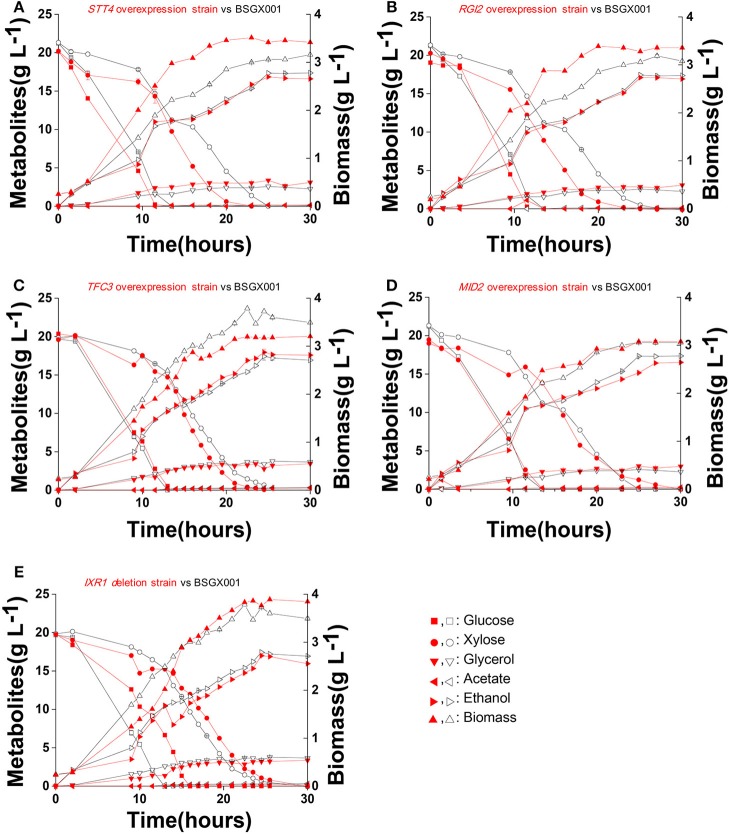
Fermentation characteristics of strains overexpressing *STT4, RGI2, TFC3*, and *MID2* or deleting *IXR1* under anaerobic conditions. Cells were cultured in bioreactors at 30 °C, pH 5.5 with 0.1 vvm nitrogen and stirring at 200 rpm. **(A)** *STT4* overexpression strain vs. BSGX001. **(B)** *RGI2* overexpression strain vs. BSGX001. **(C)** *TFC3* overexpression strain vs. BSGX001. **(D)** *MID2* overexpression strain vs. BSGX001. **(E)** *IXR1 d*eletion strain vs. BSGX001. Symbols: ■, glucose; ∙, xylose; ▾, glycerol; ◂, acetate; ▸, ethanol; ▴, biomass. The red solid symbols are the test strains; the black hollow symbols are the control strain BSGX001. All the data represent the mean value of independent triplicate tests.

The level of available oxygen has an impact on the xylose fermentation characteristics (Salusjarvi et al., 2008; Souto-Maior et al., 2009). The xylose utilization of strains overexpressing *STT4, RGI2, TFC3*, and *MID2*, or deleting *IXR1*, which showed the highest positive effect on xylose utilization in shake flask fermentations, were further evaluated in bioreactors under anaerobic conditions. Their glucose-xylose co-fermentation characteristics are shown in Figure 4 and Table 4. Overexpressing *STT4* and *RGI2* increased the specific consumption rate of xylose (r_xylose_) of the strain by 36.9 and 29.7% in the GX stage, respectively. Although the specific production rate of ethanol (r_ethanol_) and the ethanol yields (Y_ethanol_) did not increase, the fermentation time was shortened (Figures 4A,B). Overexpressing *TFC3* increased the r_xylose_ and r_ethanol_ by 42.8 and 32.5%, respectively, and this also shortened the fermentation time (Figure 4C). However, overexpression of *MID2* or deletion of *IXR1* did not yield positive effects on xylose utilization under anaerobic conditions, which indicated that oxygen levels played an important role in the strain utilization of xylose.

**Table 4:** The characteristics of anaerobic fermentation of strains overexpressing *STT4, RGI2, TFC3, MID2*, and deleting *IXR1*.

**Strains**	**μ^a^**	**${\text{r}}_{\text{xylose}}^{\text{b}}$ (g g^**−1**^ DCW h^**−1**^)**	**${\text{r}}_{\text{xylose}}^{\text{b}}$ (g g^**−1**^ DCW h^**−1**^)**	**${\text{Y}}_{\text{ethanolc}}^{\text{c}}$ (g g^**−1**^ sugars)**
BSGX001	0.170 ± 0.001	0.407 ± 0.005	0.191 ± 0.002	0.400 ± 0.002
*STT4*	0.185 ± 0.002^*^	0.557 ± 0.003^*^	0.191 ± 0.002^*^	0.396 ± 0.003
*RGI2*	0.208 ± 0.001^*^	0.528 ± 0.000^*^	0.182 ± 0.004^*^	0.400 ± 0.005
*TFC3*	0.165 ± 0.000^*^	0.581 ± 0.002^*^	0.253 ± 0.003^*^	0.400 ± 0.006
*MID2*	0.191 ± 0.001^*^	0.486 ± 0.002^*^	0.185 ± 0.002^*^	0.360 ± 0.003
*Ixr1*Δ	0.150 ± 0.002^*^	0.381 ± 0.003^*^	0.188 ± 0.001	0.402 ± 0.004

*All the data are the mean value ± standard deviation of independent triplicate tests. Cells were cultured in bioreactors at 30°C and pH 5.5, and a stirring speed of 200 rpm. The anaerobic condition was maintained by sparking nitrogen into the bioreactors with a speed of 0.1 vvm*.

* *p < 0.05*.

a *The specific growth rates (μ) were calculated from the data on the glucose consumption phase in the glucose and xylose co-fermentation*.

*^b^The specific consumption rates of xylose/ethanol (r_xylose_/r_ethanol_) were calculated from the data on the xylose consumption phase in the GX stage*.

*^c^The ethanol yields (Y_ethanol_) of total sugars that strain consumed*.

## Discussion

Despite the large amount of xylose present in many feedstocks that are commonly used in fermentation cultures in bio-industrial manufacturing, our basic understanding of xylose utilization by *S. cerevisiae* is limited. This lack of understanding has hindered rationally informed strategies for further improving recombinant *S. cerevisiae* strains to efficiently utilize this abundant carbon source. Understanding the regulatory networks controlling xylose metabolism will almost certainly inform and encourage rational engineering work focused on fully utilizing the mixed sugars in lignocellulosic hydrolysates. In the present study, we investigated the functional significance of how *THI2* deletion promotes xylose utilization. We found that *THI2* positively affected xylose utilization by downregulating the cell cycle-related gene *CIP1* and the stress response-related gene *IXR1*; by upregulating the stress response-related genes *MID2, STT4, CDC42, ECM22, BDH2*, and *CSC1*; and by upregulating the cell viability-related genes *GPN3* and *TFC3*. These results reconfirm the findings of several previous studies that have shown xylose utilization is related to stress responses, and expression of stress-resistant genes affects the xylose metabolism in engineered *S. cerevisiae* (Cheng et al., 2018; Cunha et al., 2018).

Furthermore, we found that deletion of *THI2* increased xylose metabolism in the GX stage by regulating genes involved in the cell cycle, stress tolerance, and cell viability. Notably, these regulatory targets of Thi2p in the GX stage were apparently very different from its targets when cells are cultured in glucose (e.g., eponymous thiamine biosynthetic genes and some ribosomal protein genes) (Hu et al., 2007). A comparison of these results suggests that the function of Thi2p depends on the carbon source available, a phenomenon that has been observed for some other yeast TFs (Bergenholm et al., 2018). Further modeling investigations that are directed towards optimization and engineering through disruption of TFs, such as transcriptome engineering (Michael et al., 2016), should not ignore this important difference.

In addition, our work demonstrated that overexpression of *STT4, RGI2*, and *TFC3* increased xylose consumption rates in both aerobic and anaerobic fermentation. Specifically, these genetically modified strains increased the r_xylose_ and shorted the overall fermentation time, a finding which has significance for production practices focused on improving economic efficiency. Additionally, we found that although overexpression of *MID2* or deletion of *IXR1* increased xylose consumption in aerobic fermentation, this effect was not seen in anaerobic fermentation. These results are not simply due to respiration, since the respiratory chain of our strains was blocked via deletion of *COX4*. The work of Myers et al. (2019) clearly showed large transcriptional changes to *S. cerevisiae* as it enters anaerobiosis in glucose or xylose. Future work resolving the apparent discrepancies between their transcriptome work and our findings for specific genetically manipulated strains will almost certainly reveal clues to deepen our understanding of how oxygen availability, beyond its role in respiration, impacts xylose metabolism in yeast and other organisms. Moreover, the stricter controlled pH condition in bioreactors than in shake flasks could also be a reason.

In summary, xylose has long been considered to be only a semi-fermentable carbon source for *S. cerevisiae* (Salusjarvi et al., 2008; Souto-Maior et al., 2009), and the post-glucose effect is obviously involved in shifting between carbon sources. However, this phenomenon has not been fully appreciated or understood to date. In this context, our molecular investigation of the global impacts of altering the regulatory networks controlling xylose utilization during the GX stage significantly advance our basic understanding of the mechanisms underlying such carbon source shift. Our results provide an initial proof-of-concept demonstration for new strategies to control and overcome inefficiencies for the exploitation of xylose as a carbon source in industrial biotechnology.

## Data Availability

The raw data from transcriptional analysis and processed data of genes with significant differences between the *thi2*Δ strain and the parent strain in the GX stage are presented in the NCBI Gene Expression Omnibus database (GEO accession number: GSE119333).

## Author Contributions

YS and XB conceived the original research plan. SW, PB, YL, MY, and JM designed and performed the experiments. SW, YS, XB, and JH analyzed the data. SW, YS, WL, and XB wrote and revised the manuscript. All authors read and approved the final manuscript.

### Conflict of Interest Statement

The authors declare that the research was conducted in the absence of any commercial or financial relationships that could be construed as a potential conflict of interest.

## Abbreviations

CWI: cell wall integrity
DCW: dry cell weight
GEO: Gene Expression Omnibus database
GO: Gene Ontology
LB: Luria-Bertani
PPP: pentose phosphate pathway
qPCR: Quantitative Polymerase Chain Reaction
SC: synthetic complete drop-out
SC-Ura: synthetic complete drop-out uracil
TFs: transcription factors
XI: xylose isomerase.
