# Supplementary material for: A Thi2p Regulatory Network Controls the Post-glucose Effect of Xylose Utilization in Saccharomyces cerevisiae
Source: Front Microbiol. 2019 Jul 17;10:1649. doi: 10.3389/fmicb.2019.01649 (PMC6660263; doi:10.3389/fmicb.2019.01649)
Supplement: Supplementary file 1 [file Table_1.DOC]

**Table S1 The primers used in this study.**

| **Name** | **Sequence (5' → 3')** |
| --- | --- |
| *btn2-1-up* | TCTCATTGACAGGTATTCTC |
| *btn2-1-dn* | CAGCGTACGAAGCTTCAGCTGAATTGAATATGGAAAACATTTC |
| *btn2-2-up* | GACCCCATTACAATATAGAAATGTTTTCCATATTCAATTCAGCTGAAGCTTCGTACGCTG |
| *btn2-2-dn* | TCACTGTTTATACTTTCATTATCACTTTCTGCTGAACCAGCGACTCACTATAGGGAGACC |
| *btn2-3-up* | GGTCTCCCTATAGTGAGTCGCTGGTTCAGCAGAAAGTGATAATG |
| *btn2-3-dn* | GGGAGTATGTATTATCACCC |
| *yzbtn2-up* | TCTCATTGACAGGTATTCTC |
| *yzbtn2-dn* | TTTCTACTTTACTGTTGCCC |
| *Cip1-1-up* | CTTAGTAAAGCGAACTAGAACCAGTTTAATAGGATATAGAAGCTGAAGCTTCGTACGCTG |
| *Cip1-1-dn* | ATAGGTCATGTATTGTGTGCTCCTGACTGTTCGGCACTATCGACTCACTATAGGGAGACC |
| *Cip1-2-up* | TGTATTTTCCTCCCCCTAGCTTCAACCTGTAATTCCCTTCTTAGTAAAGCGAACTAGAAC |
| *Cip1-2-dn* | AAGAGAAAGTGTAATAGAATTTCATTAAGATAGTGTCATTATAGGTCATGTATTGTGTGC |
| *Yzcip1-up* | AATGCTATTATAGAAGCGTG |
| *Yzcip1-dn* | CAACCAGAAAATGGAGAATG |
| *Dla3-up* | ATGGTGACCGTGGTGGCGGAGACATTGACGAAAGAGTCCTAGCTGAAGCTTCGTACGCTG |
| *Dla3-dn* | TTAGATGATAATACAAACGTCGCCATCGCTGTAGTGTTCTCGACTCACTATAGGGAGACC |
| *Hom3-up* | CATGCCAATGGATTTCCAACCTACATCAAGTCATTCGAACAGCTGAAGCTTCGTACGCTG |
| *Hom3-dn* | AGGATGGTGAATATTTGAGCTAGGAAACCATGGGATAGGGCGACTCACTATAGGGAGACC |
| *Ixr1-up* | ATGAACACCGGTATCTCGCCCAAACAGGACGACGCCTCTAAGCTGAAGCTTCGTACGCTG |
| *Ixr1-dn* | TCAGGGAATTGTTGAAGCAACTCATTTCTTATGGACATAGCGACTCACTATAGGGAGACC |
| *Mga1-up* | ATGCAACCTAAAACTTTCGTCCACCAACTTCATGCAATTCAGCTGAAGCTTCGTACGCTG |
| *Mga1-dn* | ATTATTACAAACTCCTAAAGCAGGTGAGTTAGAAGCAGAGCGACTCACTATAGGGAGACC |
| *Prm7-up* | ATGTATCGTACAAGAAGCTCTGACGAGGTAACCACATCAAAGCTGAAGCTTCGTACGCTG |
| *Prm7-dn* | GTCTAGAAAGGCTTAGACTTTCCGACGCAATTGGAATGCCCGACTCACTATAGGGAGACC |
| *Rrt5-up* | GAAGTATAAGGCCTCACATAAGCATACAAACAAGCCCGCAAGCTGAAGCTTCGTACGCTG |
| *Rrt5-dn* | GTTCTCACTGTTTATACCCCCTTTTCAATTTTATAAAGATCGACTCACTATAGGGAGACC |
| *Ydr246w-A-up* | CTGCGACGGATCCTCTGCATCCCAACACCAGCGATAGAAAAGCTGAAGCTTCGTACGCTG |
| *Ydr246w-A-dn* | AAAGTAGGATCGACTACCCCTTTTGGGTTACACTTTGGTACGACTCACTATAGGGAGACC |
| *Ygl015c-up* | GGTATTGGACAGATATAATTCACAAATCACAATTCACTCCAGCTGAAGCTTCGTACGCTG |
| *Ygl015c-dn* | TGTGCTTTAAAGATAGTAGCAGTATATATGTGTGTAAAAGCGACTCACTATAGGGAGACC |
| *Yor338w-up* | ATGCTAGATAACATGCAATTTCATTCTCCAGCACCGGAACAGCTGAAGCTTCGTACGCTG |
| *Yor338w-dn* | TTACAAATACTTTTCAAAATGTTTATCTTGTAACCAACCTCGACTCACTATAGGGAGACC |
| *Bsc1-up* | CAACTGGCACATGAGCCAACAGAATATACTACATTATGATAGCTGAAGCTTCGTACGCTG |
| *Bsc1-dn* | CGCTTGAACTAGTTGGGTCTTCTGAACTTGAACTTACTACCGACTCACTATAGGGAGACC |
| *ARV1-UP* | ATAAGAATGCGGCCGCATGATTTGCATAACGTGCATG |
| *ARV1-DN* | CCTTAATTAATTATAACAATAAATAAGTTCCTGAC |
| *ECI1-UP* | ATAAGAATGCGGCCGCATGTCGCAAGAAATTAGGCAAAATG |
| *ECI1-DN* | CCTTAATTAATCATAAACGATGCTTCCTTTG |
| *FAR10-UP* | CTAATCTAAGTTTTAATTACAAGCGGCCGCATGACTGGTCCTGGACCTGAAATAAATAAG |
| *FAR10-DN* | CCTTGTAATCCATCGATACTAGTGCGGCCGCCTAGTTGGGGGAAAGGATCTTG |
| *PEX22-UP* | CAATCTAATCTAAGTTTTAATTACAAGCGGCCGCATGCCACCACCATCAAGAAGTAG |
| *PEX22-DN* | CCTTGTAATCCATCGATACTAGTGCGGCCGCTTAATTGCATAAAGTGTCAATCAGC |
| *MID2-UP* | CAATCTAATCTAAGTTTTAATTACAAGCGGCCGCATGTTGTCTTTCACAACCAAG |
| *MID2-DN* | CATCCTTGTAATCCATCGATACTAGTGCGGCCGCTTAATAATTTCGTGGTGATAATTCG |
| *BDH2-UP* | ATAAGAATGCGGCCGCATGAGAGCCTTAGCGTATTTC |
| *BDH2-DN* | CCTTAATTAATCATGTGTGACGCAGTTTAG |
| *BOL1-UP* | ATAAGAATGCGGCCGCATGTTCAAGAGAGCAATGAG |
| *BOL1-DN* | CCTTAATTAACTATTTCGCTTTGGATTCATAC |
| *COQ11-UP* | ATAAGAATGCGGCCGCATGATACCAAAGCTTATAGTTTTTGG |
| *COQ11-DN* | CCTTAATTAATTATGCTTTAAGTATTTCCTCAAG |
| *CSC1-UP* | ATAAGAATGCGGCCGCATGACATCATATATCGAAAGGCTC |
| *CSC1-DN* | CCTTAATTAATCAATCCAACATCGGACCCTC |
| *DBP9-UP* | ATAAGAATGCGGCCGCATGAGCTATGAGAAAAAGTC |
| *DBP9-DN* | CCTTAATTAATCATTTGAAGTTCTTCAACG |
| *ECM22-UP* | ATAAGAATGCGGCCGCATGACATCCGATGATGGGAATG |
| *ECM22-DN* | CCTTAATTAATTACATAAAAGCTGAAAAGTTTGTAGTG |
| *GPB2-UP* | ATAAGAATGCGGCCGCATGGAAATTTCCAGTTCACC |
| *GPB2-DN* | CCTTAATTAATTATGCACTAGGATTTACACTAGG |
| *GPN3-UP* | ATAAGAATGCGGCCGCATGTCTCGCGTTGGTGTCATG |
| *GPN3-DN* | CCTTAATTAACTATTCTTCGACATCTATTTGGTC |
| *MRPL15-UP* | AAATATGCGGCCGCATGGAAAATAGCATGATGTTTATATCG |
| *MRPL15-DN* | GACCTTAATTAATCACAGAAATGCTTTATTTTCC |
| *PHO11-UP* | ATAAGAATGCGGCCGCATGTTGAAGTCAGCCGTTTATTC |
| *PHO11-DN* | CCTTAATTAATTACTGTTTTAATAAAGTGTCGTTG |
| *PIG1-UP* | ATAAGAATGCGGCCGCATGCCTTACAGCCATGGTAAG |
| *PIG1-DN* | CCTTAATTAATTAGGGTGACGGCGACTTATAAAAATAATC |
| *RBG1-UP* | AAATATGCGGCCGCATGTCTACTACAGTTGAAAAAATC |
| *RBG1-DN* | GACCTTAATTAATCACTTTTTCAAGATGGTAAC |
| *MCM5-UP* | ATAAGAATGCGGCCGCATGTCATTTGATAGACCGG |
| *MCM5-DN* | CCTTAATTAATCATACACCACTTCTGTAAATATTC |
| *RGI2-UP* | ATAAGAATGCGGCCGCATGACGAAAAAGGATAAGAAAG |
| *RGI2-DN* | CCTTAATTAATTAGATGAGTGGCTGCAAGGG |
| *RPS25B-UP* | ATAAGAATGCGGCCGCATGCCTCCAAAGCAACAATTG |
| *RPS25B-DN* | CCTTAATTAATTATTCGGAAGCAGCAGCTC |
| *RPS28B-UP* | ATAAGAATGCGGCCGCATGGATTCTAAGACCCCAGTC |
| *RPS28B-DN* | CCTTAATTAATTAACGCAAACGACGAGCTTC |
| *SFH1-UP* | ATAAGAATGCGGCCGCATGTCGCACCAAAACCAGC |
| *SFH1-DN* | CCTTAATTAACTACATTCTCATTGTGGTTTC |
| *STT4-UP* | ATAAGAATGCGGCCGCATGAGATTTACCAGAGGATTG |
| *STT4-DN* | CCTTAATTAATCAGTACGGAATGCCATTTG |
| *MAK16-UP* | AAATATGCGGCCGCATGTCCGACGAAATTGTTTG |
| *MAK16-DN* | GACCTTAATTAATTATTGTGCCACTTCTTGCTC |
| *THI7-UP* | ATAAGAATGCGGCCGCATGAGTTTCGGTAGTAAAGTC |
| *THI7-DN* | CCTTAATTAACTAAGCAGCTTTTTCACTGG |
| *TFC3-UP* | ATAAGAATGCGGCCGCATGGTACTGACGATTTATCC |
| *TFC3-DN* | CCTTAATTAATTATGTAGATTCATATATAGAATACC |
| *RPL38-UP* | ATAAGAATGCGGCCGCATGGCTAGAGAAATCACCG |
| *RPL38-DN* | CCTTAATTAATTATAATCTGTTAACCTTCAAAGTTG |
| *RPPO-UP* | AAATATGCGGCCGCATGGGAGGCATTCGTGAAAAG |
| *RPPO-DN* | GACCTTAATTAATTAATCGAATAAACCGAAACCC |
| *CDC42-UP* | ATAAGAATGCGGCCGCATGCAAACGCTAAAGTGTG |
| *CDC42-DN* | CCTTAATTAACTACAAAATTGCACATTTTTTAC |
| *BOP2-UP* | ATAAGAATGCGGCCGCATGGTTGCCGCTTTAACGTATTTG |
| *BOP2-DN* | CCTTAATTAATCAAACCACGACAAACTTCTGTC |
| *POP5-UP* | ATAAGAATGCGGCCGCATGGTACGTTTAAAAAGTAGATATATC |
| *POP5-DN* | CCTTAATTAACTAATCGTCCTCGTTTTCGTTTTC |
| *UBC12-UP* | ATAAGAATGCGGCCGCATGGTACGTAGATGCAACTATATC |
| *UBC12-DN* | CCTTAATTAATCAAGGAGAAACTATGTTATCATATTTG |
